# Supplementary material for: The gut ileal mucosal virome is disturbed in patients with Crohn’s disease and exacerbates intestinal inflammation in mice
Source: Nat Commun. 2024 Feb 22;15:1638. doi: 10.1038/s41467-024-45794-y (PMC10884039; doi:10.1038/s41467-024-45794-y)
Supplement: Supplementary file 1 — Supplementary Information [file 41467_2024_45794_MOESM1_ESM.pdf]

Supplementary Figures

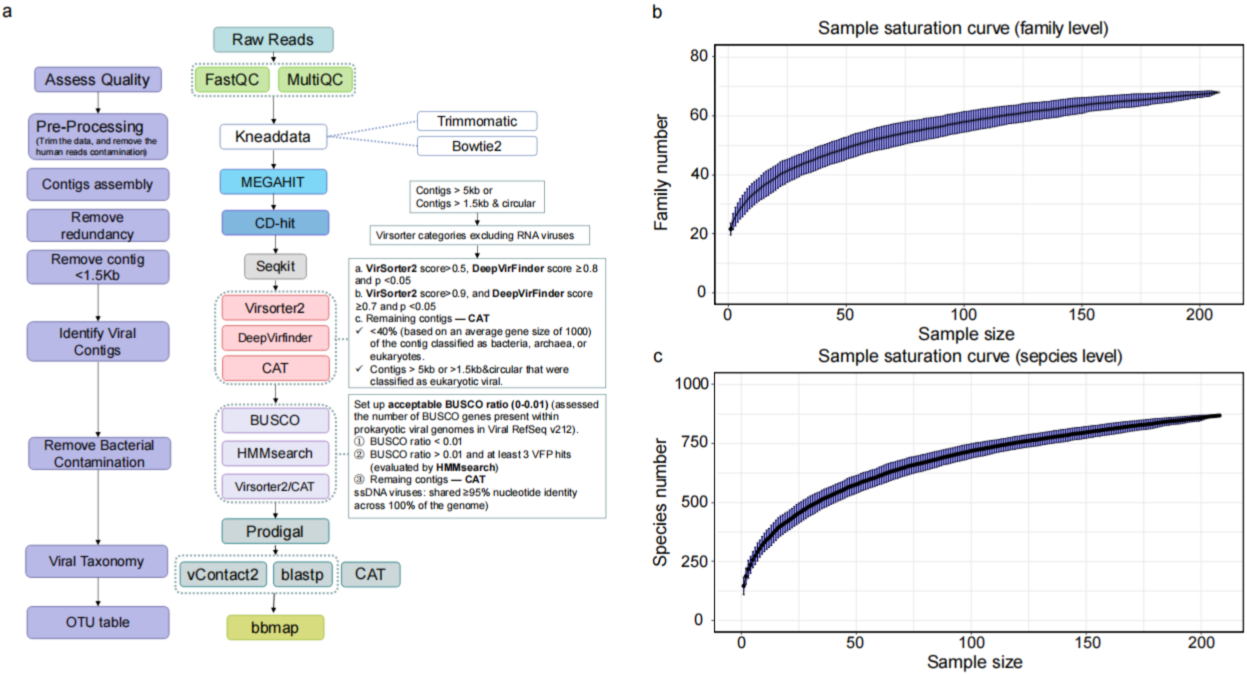

Supplementary Fig. 1 Workflow of virome analysis pipeline and sample size estimation.
(a) Pipeline and the analysis workflow for the ileal virome metagenomic data.
(b-c) Sample saturation curve at the family (b) and species (c) levels respectively.

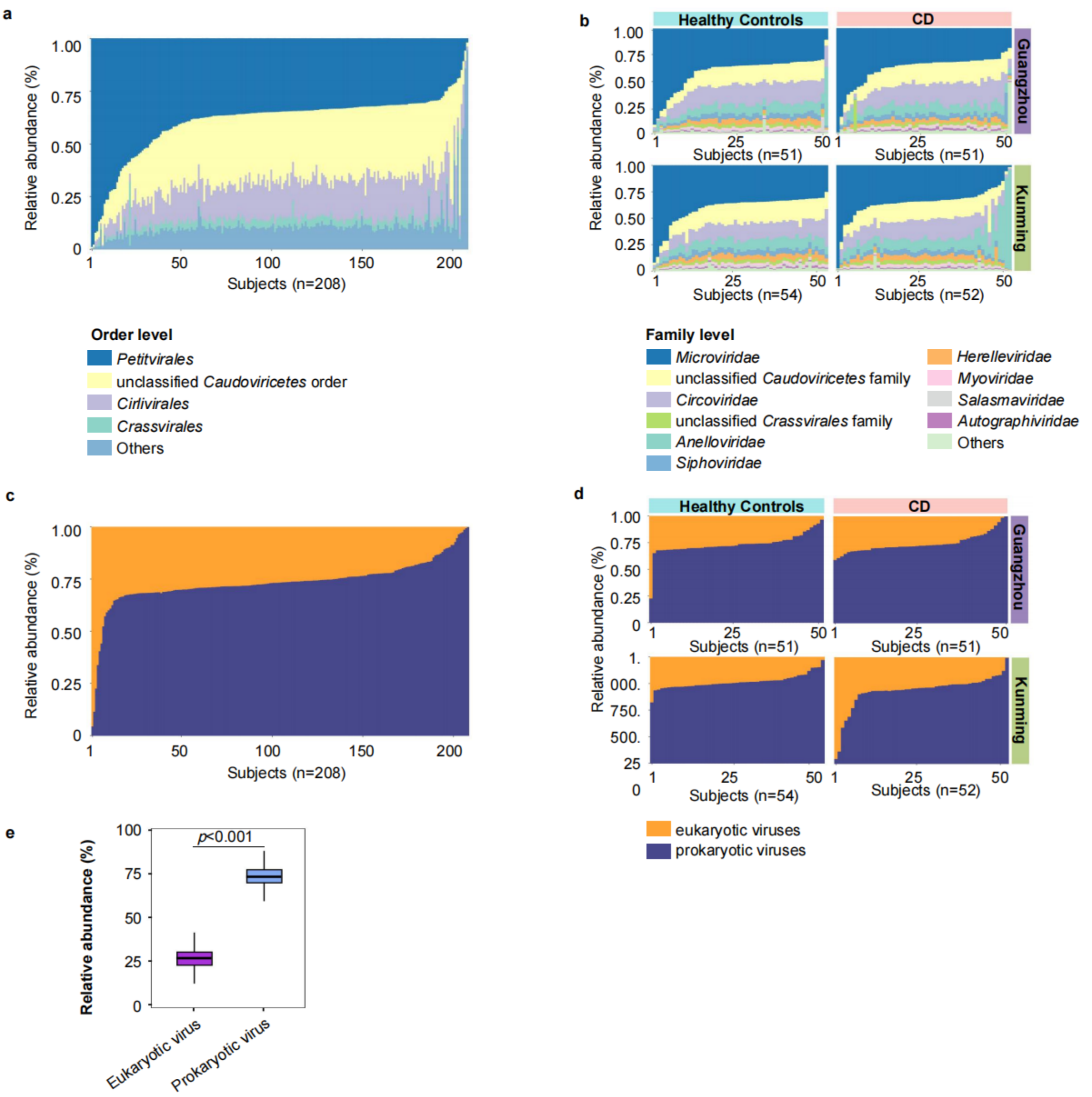

Supplementary Fig. 2 Variations in the ileal virome across study subjects.
(a) Variations in the ileal virome composition across all subjects (n=208), plottedaccording to the ranking of the relative abundance of ileal viruses at the order level.
(b) Variations in the ileal virome across all subjects at the family level, plotted with respect to HC, CD, and geographic regions (Guangzhou and Kunming).
(c) Variations in the ratio of eukaryotic viruses to prokaryotic viruses across all subjects.
(d) Variations in the ratio of eukaryotic viruses to prokaryotic viruses, plotted with respect to HC, CD, and geographic regions (Guangzhou and Kunming).
(e) Relative abundance of eukaryotic viruses and prokaryotic viruses (bacteriophages) in the ileal mucosal virome. For box plots, the boxes extend from the 1st to the 3rd quartile (25th to 75th percentiles), with the median depicted by a horizontal line. Statistical significance was determined by two-tailed *t* test.

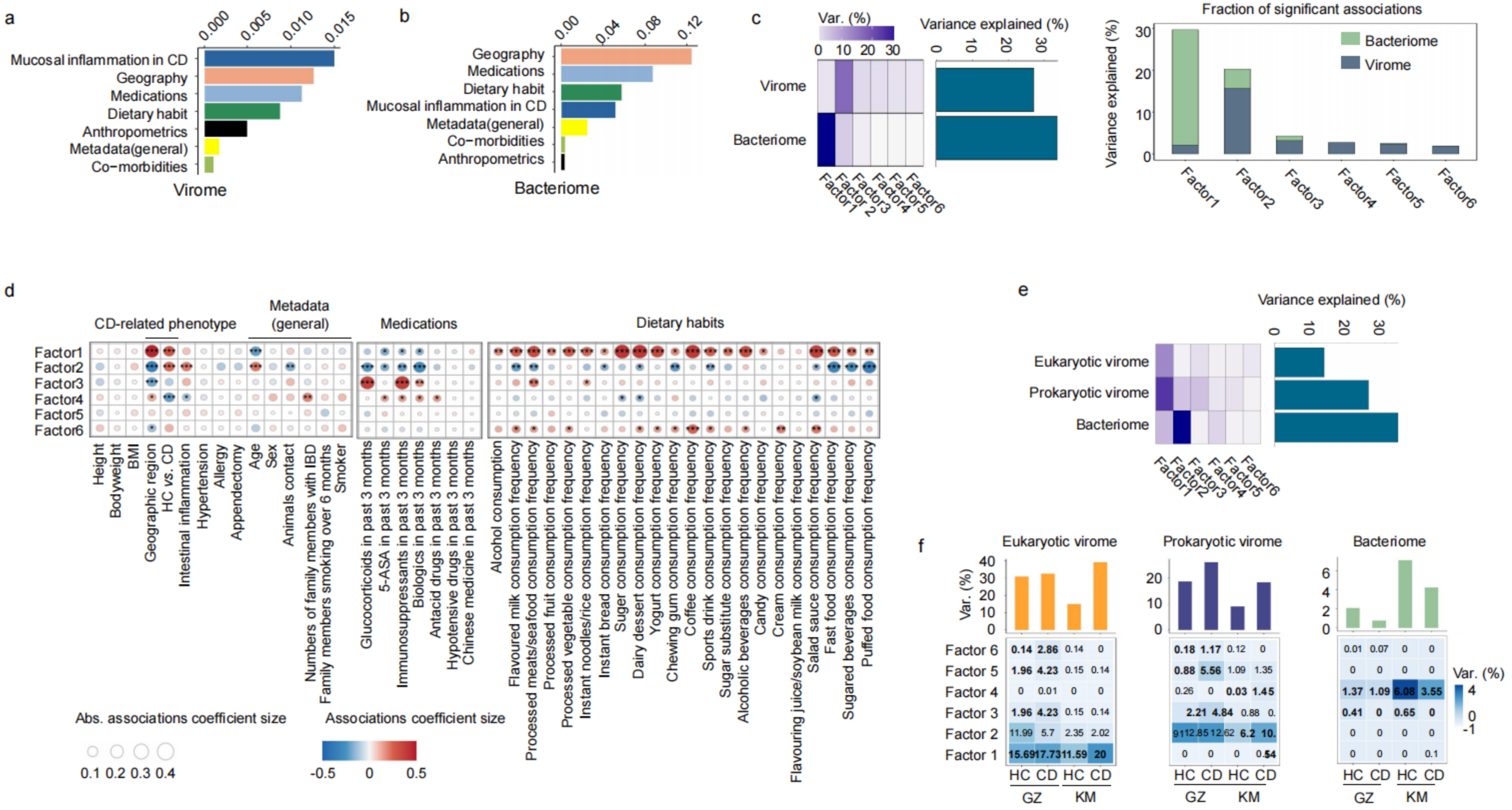

Supplementary Fig. 3 The varying microbiome characteristics and their associated metadata.
(a-b) Virome and bacteriome covariates (metadata factors in Fig. 1g and i) were pooled in predefined categories (mucosal inflammation in CD, anthropometrics, geography, medications, dietary habit, co−morbidities, and metadata [general]), followed by effect size calculation with variance partitioning analysis. Data were analysed through vegan in R.
(c) Heat map and bar plot (left panel) displayed the percentage of variance explained by 6 predominant microbiome factors across the two microbiome omics-data (virome and bacteriome), and the bar plot (right panel) detailed the respective contributions of virome and bacteriome to the variations of microbiome in each of the 6 predominant microbiome factors. Data analysis was performed by the multi-omics analysis pipeline MOFA.
(d) Bubble plot displayed the associations between the 6 predominant microbiome factors and the clinical metadata, identified by MOFA analysis. Red bubbles indicate positive correlations, while blue bubbles indicate inverse correlations. The size and shading intensity indicate the magnitude of the correlation. Significant correlations are displayed with asterisks, **p* <0.05, ***p* <0.01, ****p* <0.001.
(e) Heat map and bar plot displayed the percentage of variance explained by each microbiome factor across the three microbiome omics-data (the prokaryotic virome, the eukaryotic virome, and the bacteriome).
(f) Heat map and bar plot displayed the percentage of variances explained by each microbiome factor across the three microbiome omics-data (the prokaryotic virome, the eukaryotic virome, and the bacteriome), plotted with respect to HC, CD and geography.

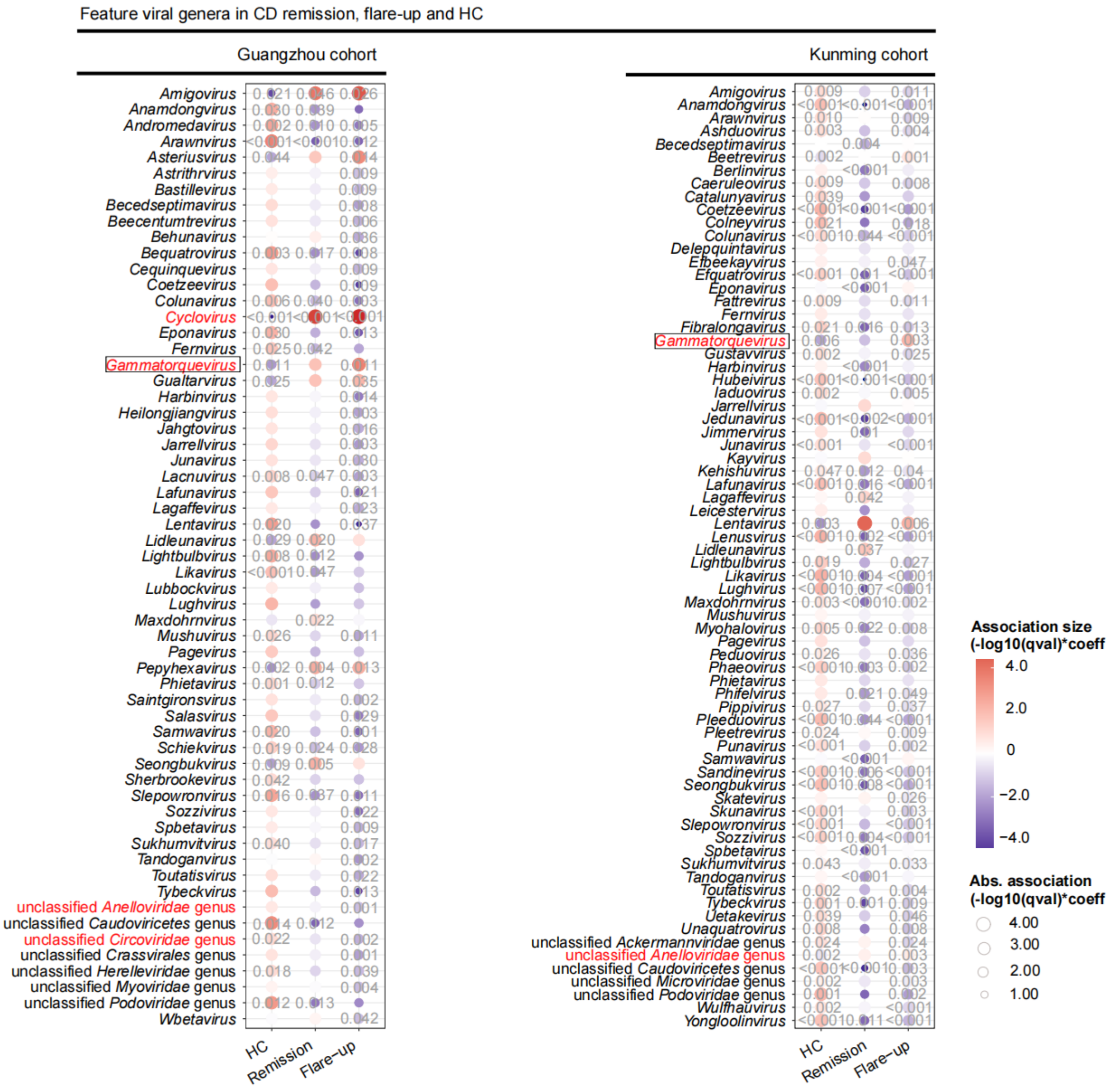

Supplementary Fig. 4 Feature viral genera different in CD remission and flare-up.
Feature genera in CD were identified by *MaAsLin2* with the Benjamini and Hochberg false discovery rate (BH-FDR) adjustment controlling for medications and dietary habit, and q <0.2 (p <0.05) was considered significant. Red bubbles indicate genera positively associated (increased in abundance) with the group of interest (HC, CD remission or flare-up), and blue bubbles indicate genera inversely associated (decreased in abundance) with the group of interest. The bubble size and shading intensity are proportional to the correlation coefficient between the viral genera and the group of interest. Viral taxa colour-coated in black denote prokaryotic viruses (bacteriophages), while those colour-coated in red denote eukaryotic viruses. Statistical significance was determined by a two-tailed multivariable association test, and adjusted by the BH-FDR adjustment.

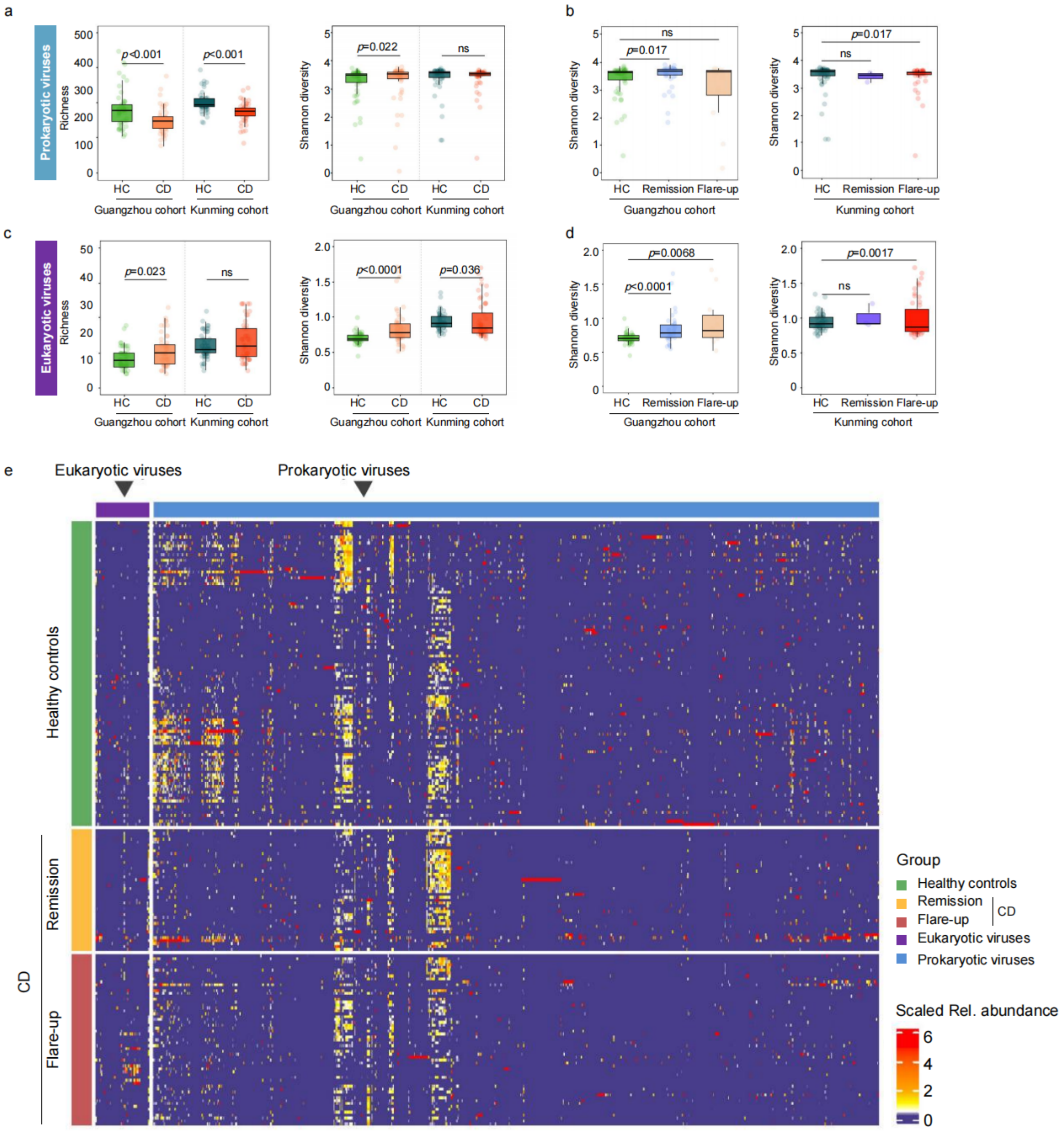

Supplementary Fig. 5 Alterations in the α diversity and abundance of ileal eukaryotic viruses and prokaryotic viruses in CD.
(a&c) The α diversity (richness or Shannon diversity) of ileal prokaryotic viral species (a) and eukaryotic viral species (c) in the Guangzhou (n=102) and Kunming (n=106) cohorts respectively, compared between HC and CD. Statistical significance was determined by two-tailed *t* test.
(b&d) The Shannon diversity of ileal prokaryotic viral species (b) and eukaryotic viral species (d) in the Guangzhou and Kunming cohorts respectively, compared between HC, CD remission and flare-up. Statistical significance was determined by the two-tailed *Mann-Whitney* test.
(e) Relative abundance heatmap of the prokaryotic viral species and eukaryotic viral species of the ileal virome in HC, CD remission and flare-up. The relative abundance of ileal viral species was normalized by Z-score transformation via *scale ()* function in R.
For box plots, the boxes extend from the 1st to the 3rd quartile (25th to 75th percentiles), with the median depicted by a horizontal line.

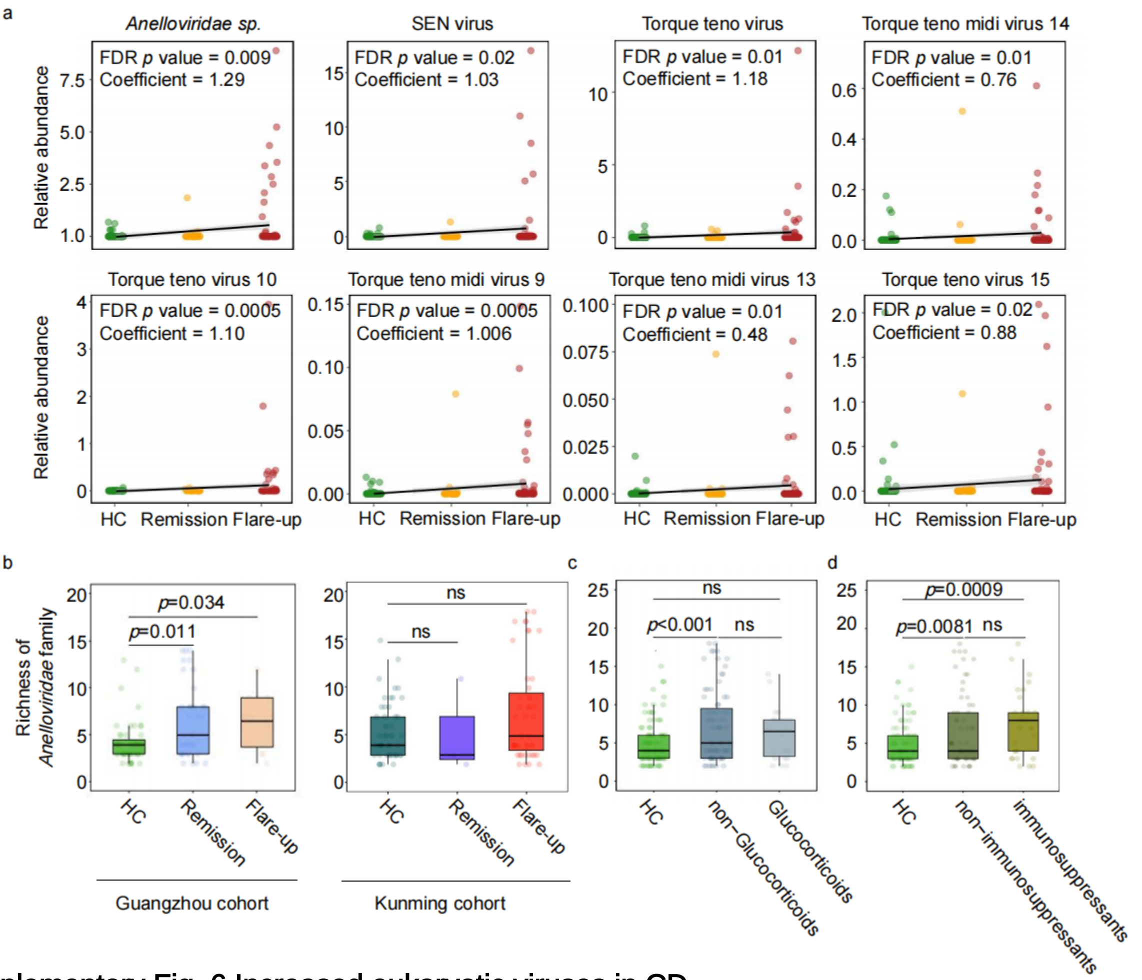

Supplementary Fig. 6 Increased eukaryotic viruses in CD.

(a) Correlation between the relative abundances of eukaryotic viral species and intestinal inflammation phenotype (n=208, HC, CD remission and flare-up). Associations were estimated via *MaAsLin2* analysis, where coefficient and false discovery rate (FDR)adjusted *p* value were determined controlling for geography, medications and dietary habit.

(b) The richness of the *Anelloviridae* family in the Guangzhou (left, n=102) and Kunming (right, n =106) cohorts, respectively, compared between HC and CD remission and flare-up. Statistical significance between HC and CD remission/flare-up was determined by the two-tailed *Mann-Whitney* test.

(C) Comparison of ileal *Anelloviridae* richness between HC (n=105) and CD patients (n=103) with or without immunosuppressants medications in the past 3 months. Statistical significance between groups was determined by the two-tailed *Mann-Whitney* test.

(D) Comparison of ileal *Anelloviridae* richness between HC and CD patients with or without glucocorticoids medications in the past 3 months. Statistical significance between groups was determined by the two-tailed *Mann-Whitney* test.

For box plots, the boxes extend from the 1st to the 3rd quartile (25th to 75th percentiles), with the median depicted by a horizontal line.

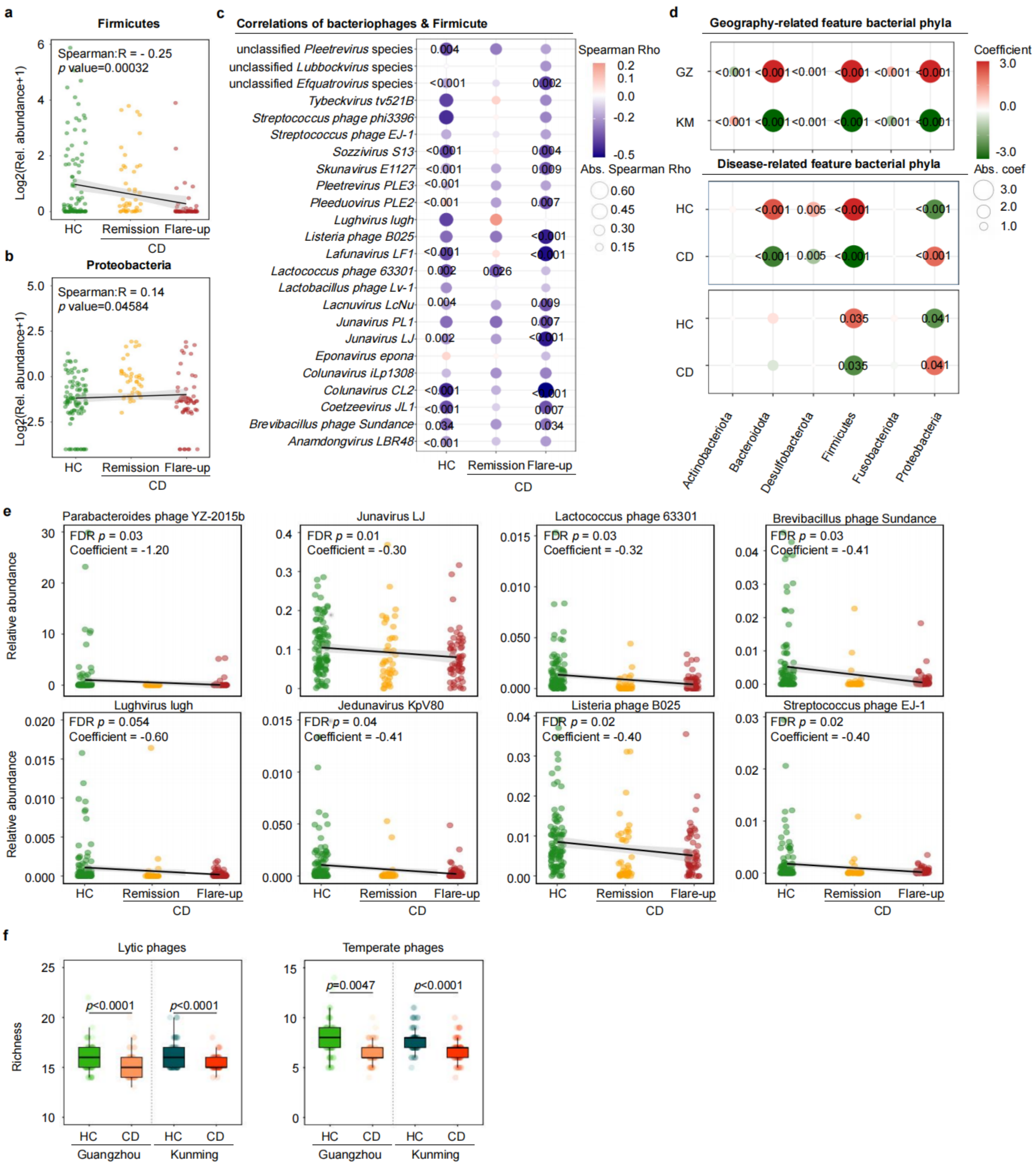

Supplementary Fig. 7 Abundance differences in bacteria and viruses with CD status and geography.
(a-b) The relative abundance of Firmicutes and Proteobacteria in the ileal mucosal bacteriome in HC, CD remission and flare-up respectively (n=208).
(c) Correlations between bacteriophages and the bacterial phylum Firmicutes, plotted in bubble plot. Spearman's correlation coefficient was calculated, while statistical significance was determi ned for all pairwise comparisons. Red bubbles indicate positive correlations, while blue bubbles in dicate inverse correlations. The size and shading intensity indicate the magnitude of the correlation.
(d) Geography-related bacterial phyla and disease (CD)-related bacterial phyla, identified by *MaAsLin2* with BH-FDR adjustment controlling for medications and dietary habit, with n=102 samples in Guangzhou and n=106 samples in Kunming cohorts respectively, and q <0.2 (p <0.05) was considered significant. GZ, Guangzhou; KM, Kunming.
(e) Correlations between the relative abundances of CD-depleted prokaryotic viral species and intestinal inflammation phenotype (HC, CD remission and flare-up). Associations were estimated via *MaAsLin2* analysis, where coefficient and FDR-adjusted p values were determined controlling for geography, medications and dietary habit.
(f) The Richness of lytic phages (f) and temperate phages (g) in the Guangzhou and Kunming cohorts respectively, compared between HC and CD.
For box plots, the boxes extend from the 1st to the 3rd quartile (25th to 75th percentiles), with the median depicted by a horizontal line. Statistical significance between CD and HC was determined by two-tailed t test.

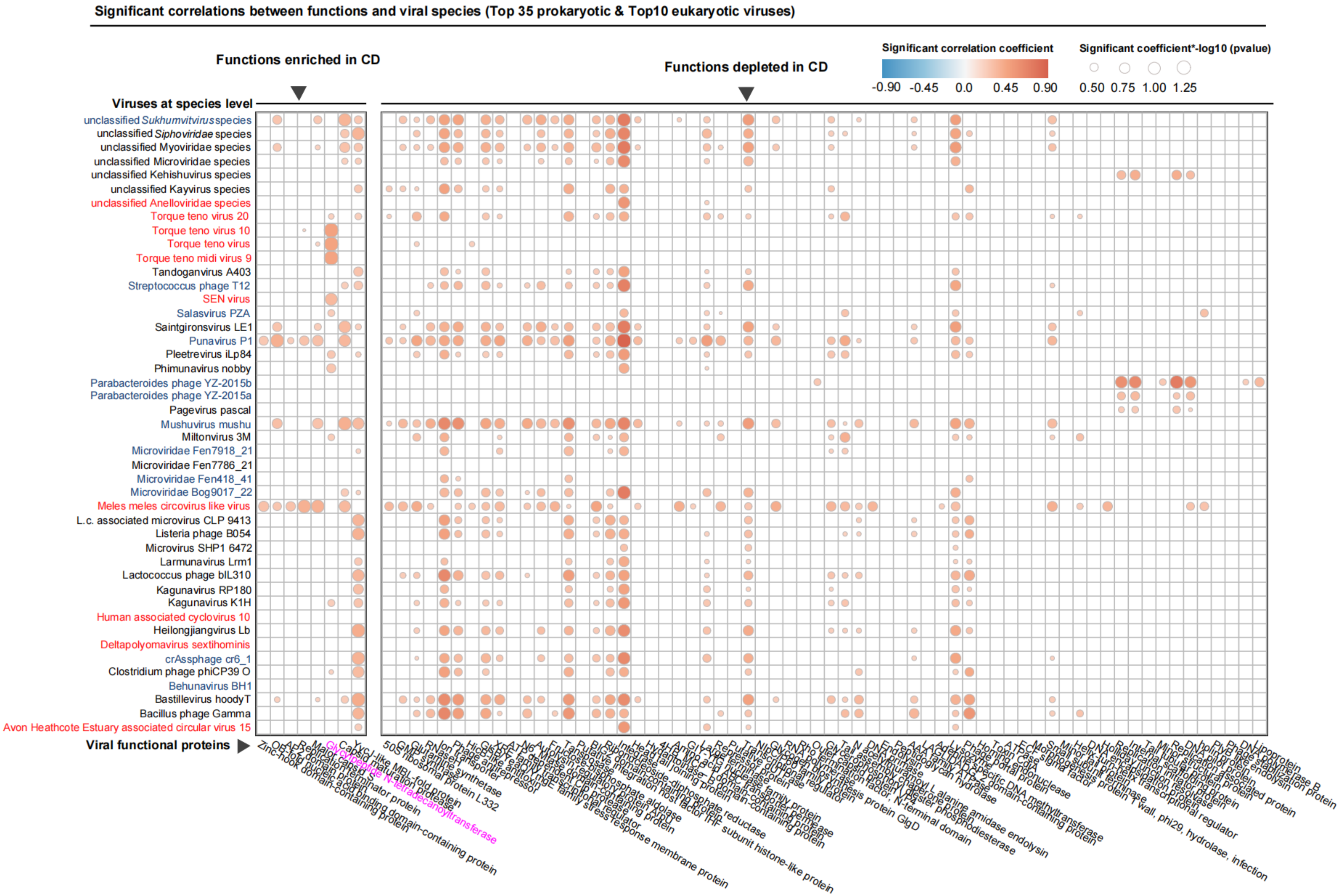

Supplementary Fig. 8 Correlations between viral functions and species.
Correlations between CD-featured viral functions (including functions enriched or depleted in CD) and the most abundant viral species (only the top 35 bacteriophages and the top 10 eukaryotic viruses were included). The correlations were calculated with *FastSpar*, and statistical significance was determined for all pairwise comparisons by a bootstrap method. Only statistically significant correlations were plotted. The size and shading intensity indicate the magnitude of the correlation. Viral taxa colour-coated in black denote prokaryotic viruses (bacteriophages), while those colour-coated in red denote eukaryotic viruses. Bacteriophages colour-coated in blue denote the species depleted in CD versus HC.

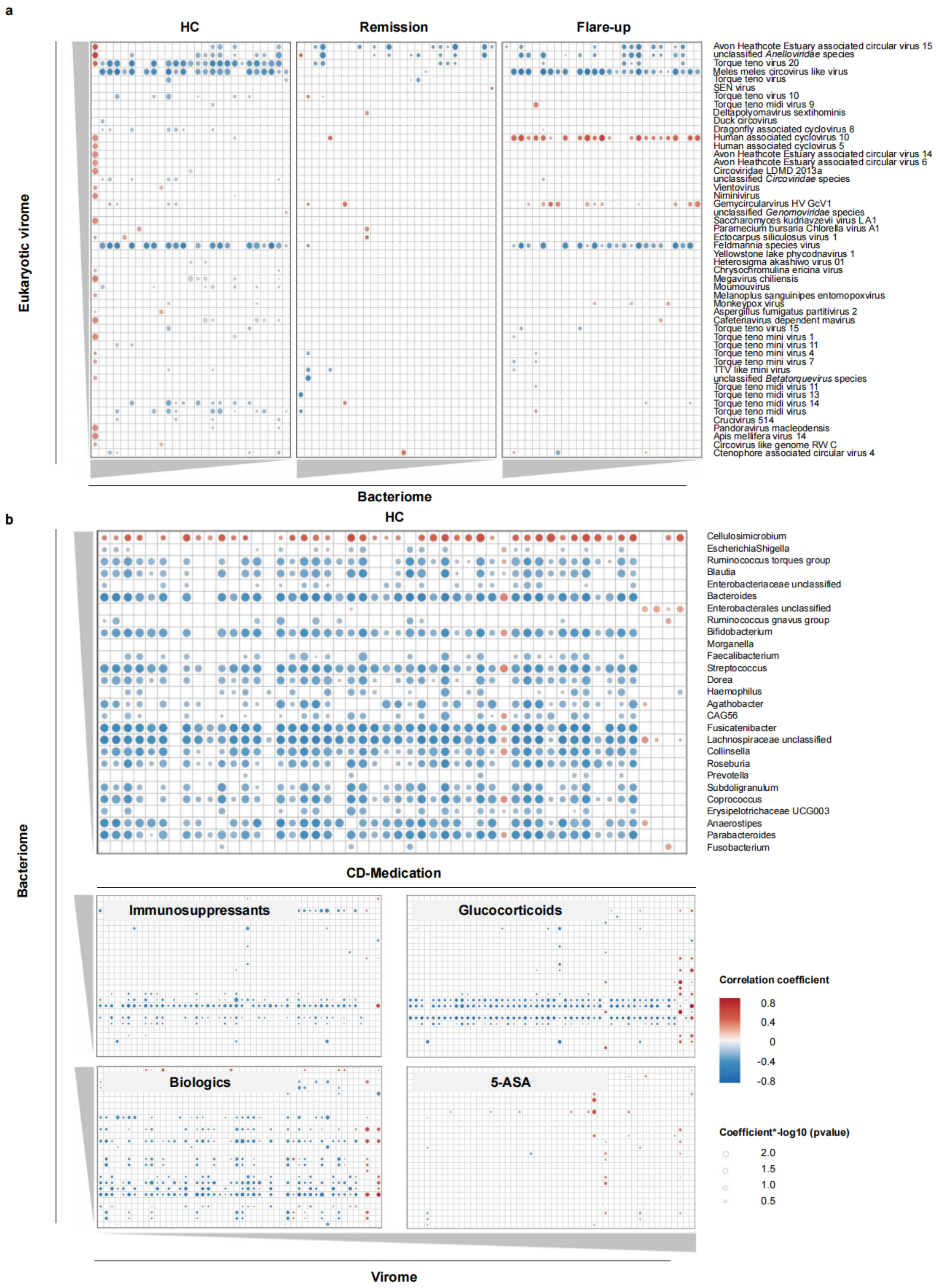

Supplementary Fig. 9 Trans-kingdom correlations between ileal eukaryotic viruses and bacteria, and the trans-kingdom correlation network between ileal phageome and bacteriome, according to CD treatment. (a) Trans-kingdom correlations between the top 50 eukaryotic viruses and the top 30 bacterial genera in HC, CD remission and flare-up. The correlations were calculated with *FastSpar*, while statistical significance was determined for all pairwise comparisons by a bootstrap method. Only statistically significant correlations were plotted. Red bubbles indicate positive correlations and blue bubbles indicate inverse correlations. The size and shading intensity indicate the magnitude of the correlation.

(b) Trans-kingdom correlation network between the top 50 bacteriophage species and the top 30 bacterial genera with respect to CD treatment (including immunosuppressants, glucocorticoids, biologics, and 5-aminosalicylic acid [5-ASA]). The correlations were calculated with *FastSpar*, while statistical significance was determined for all pairwise comparisons by a bootstrap method. Only statistically significant correlations were plotted. Red bubbles indicate positive correlations, while blue bubbles indicate inverse correlations. The size and shading intensity indicate the magnitude of the correlation.

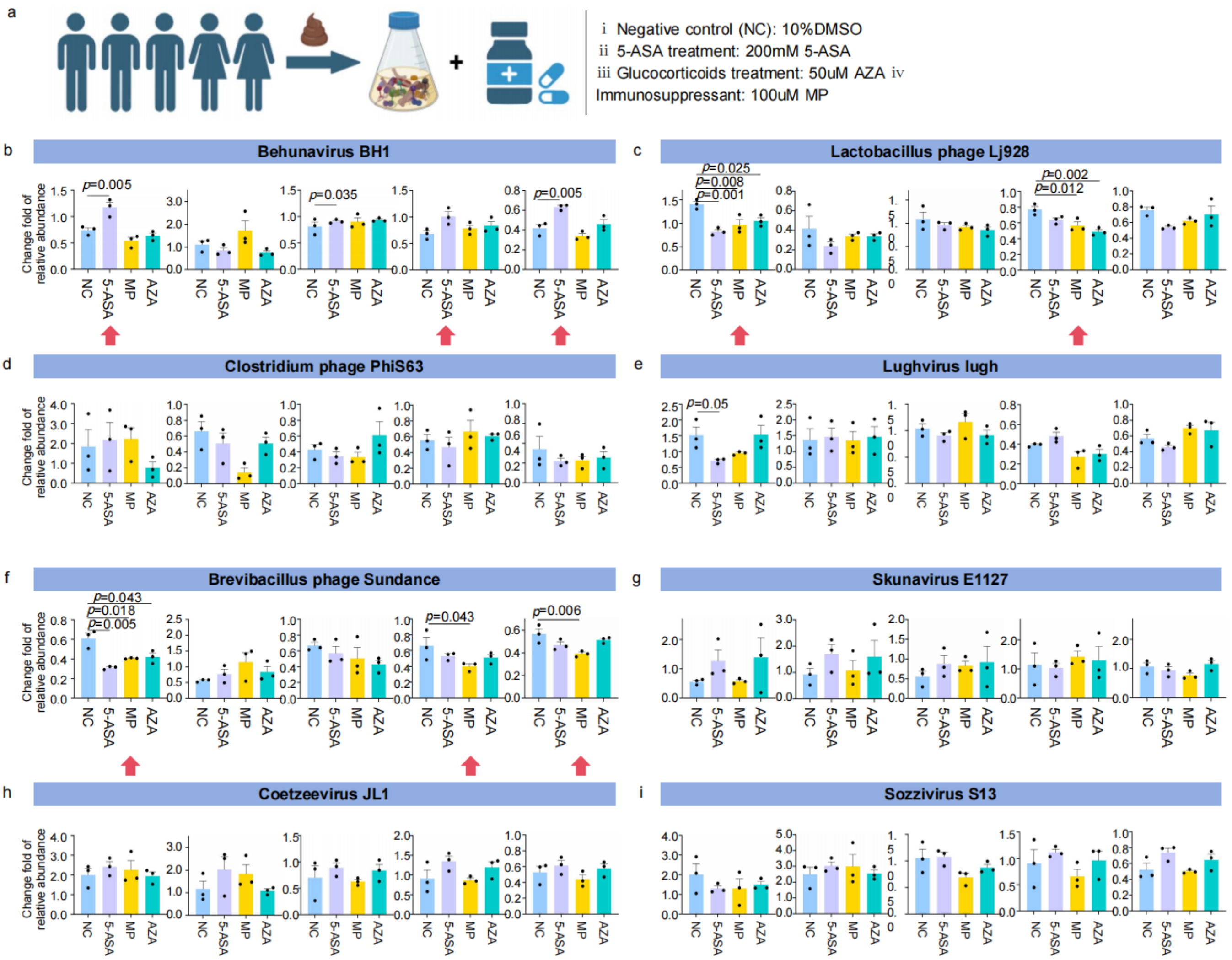

Supplementary Fig. 10 The effect of medications on the gut virome, in vitro experiments.

(a) Schematic of in vitro experiment. Faecal microbiome preparations were incubated with 200mM 5-ASA, 50uM AZA, 100uM MP, and negative control (10% DMSO) respectively, for 12 hours, followed by target phage quantification.

(b-i) Relative abundance change in target phages post medication, measured by q-PCR. Statistical significance between groups was determined by the two-tailed *Mann-Whitney* test, based on three independent experiments conducted for each faecal sample, representing as mean +/- SEM.

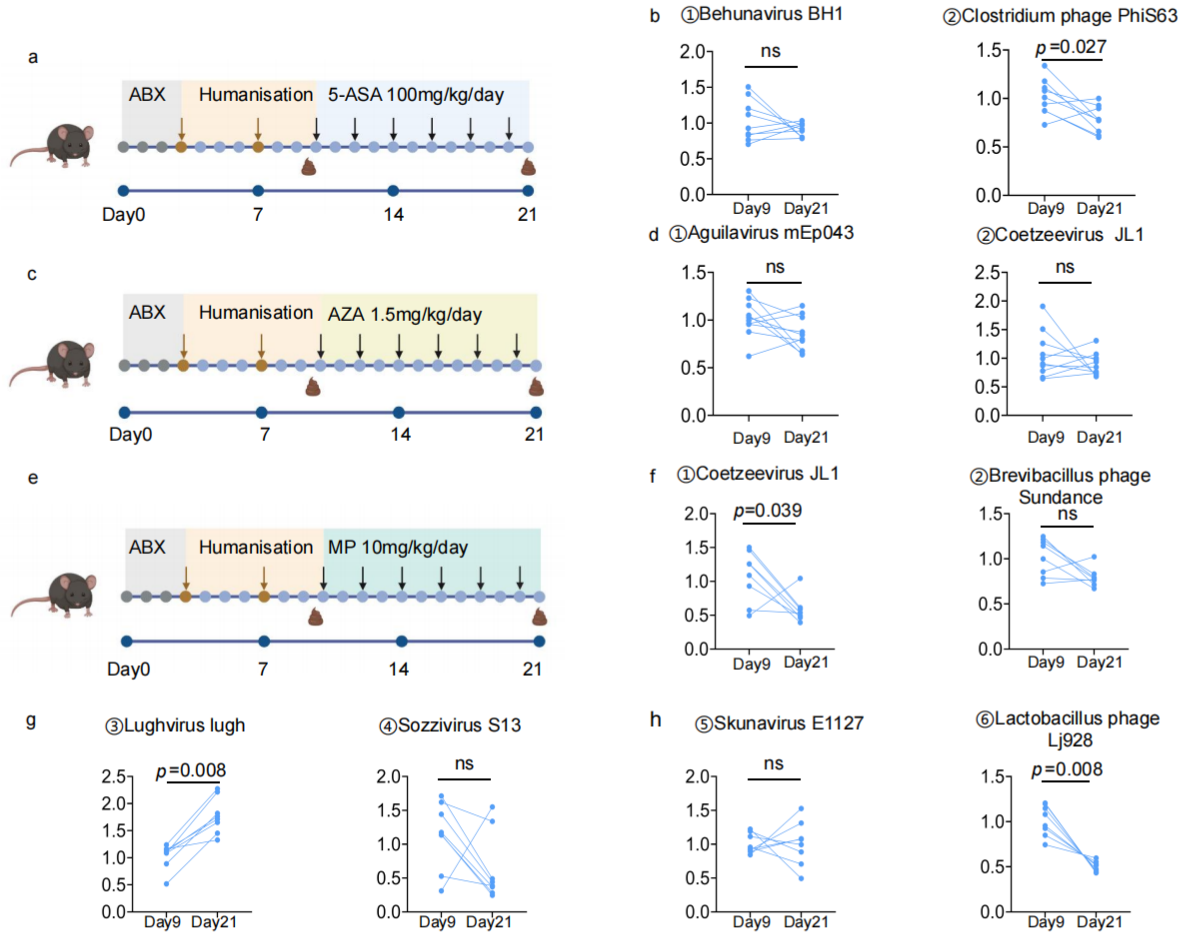

Supplementary Fig. 11 The effect of medications on the gut virome, in vivo experiments.
(a, c& e) Schematic of in vivo experiments in mice. Mice were administrated 5-ASA, AZA, and MP, respectively (n=10 each group), followed by target phage quantification.
(b, d, f& h) Relative abundance change in target phages prior to and post-medication treatment, measured by q-PCR. Statistical significance between groups was determined by the two-tailed Paired Rank-Sum test.

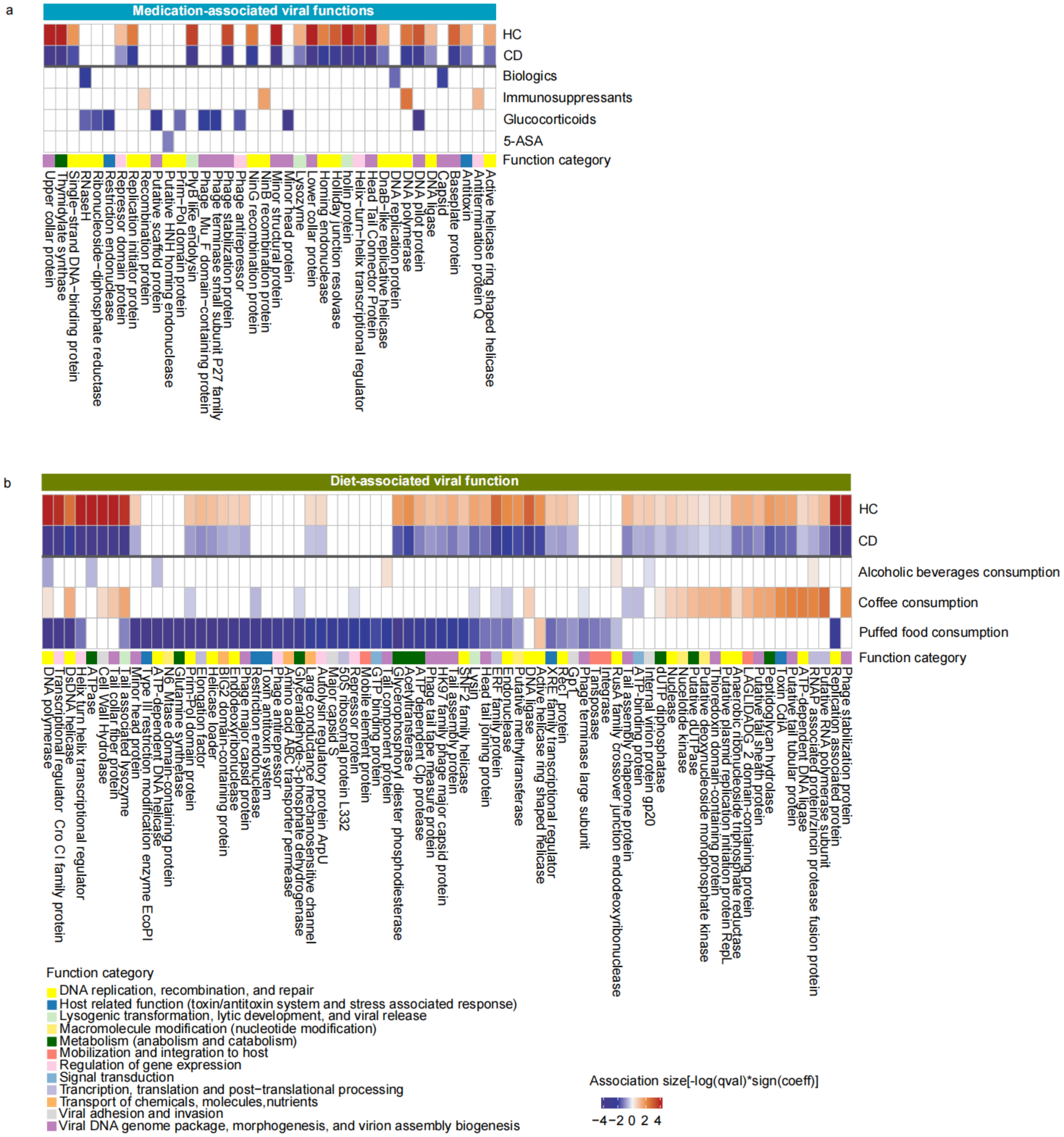

Supplementary Fig. 12 Medications- and diet-associated virome functions.
(a) Functionality alterations of the ileal virome with respect to CD treatment including immunosuppressants, glucocorticoids, biologics, and 5-ASA. The differential functions were identified via *MaAsLin2* with BH-FDR adjustment controlling for geography and dietary habit. Only the significant function modules with BH-FDR $q<0.2$ ($p<0.05$) were shown. The associations of these viral functions with CD were also plotted for comparative visualization.
(b) Function modules of the ileal virome associated with the consumption of alcoholic beverage, coffee consumption, and puffed food. The distinctive functions were identified via *MaAsLin2* with BH-FDR adjustment controlling for geography and medications. Only the significant function modules with BH-FDR $q<0.2$ ($p<0.05$) were shown.

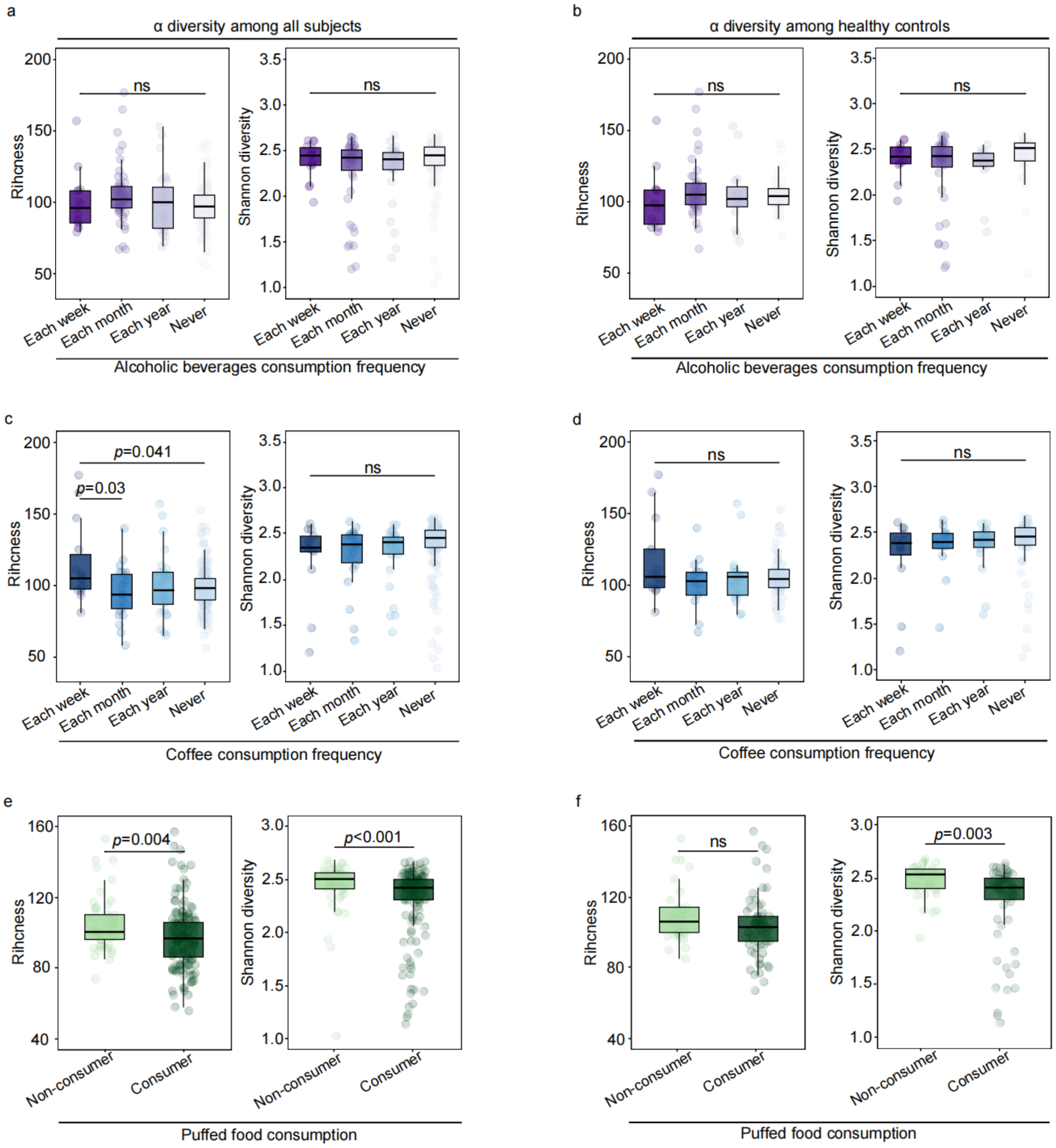

Supplementary Fig. 13 Changes in the α diversity of ileal mucosal virome in association with consumptions of alcoholic beverages, coffee, and puffed foods.

(a-b) The α diversity of the ileal virome in association with consumption frequency (never, each week, each month, and each year) of alcoholic beverages among all subjects (a, n=208) or among all healthy controls (b, n=105).

(c-d) The α diversity of the ileal virome in association with consumption frequency (never, each week, each month and each year) of coffee among all subjects (c) or among all healthy controls (d).

(e-f) The α diversity of the ileal virome in association with consumption of puffed foods among all subjects (e) or among all healthy controls (f).

For box plots, the boxes extend from the 1st to the 3rd quartile (25th to 75th percentiles), with the median depicted by a horizontal line. Statistical significance between groups was determined by the two-tailed *Mann-Whitney* test.

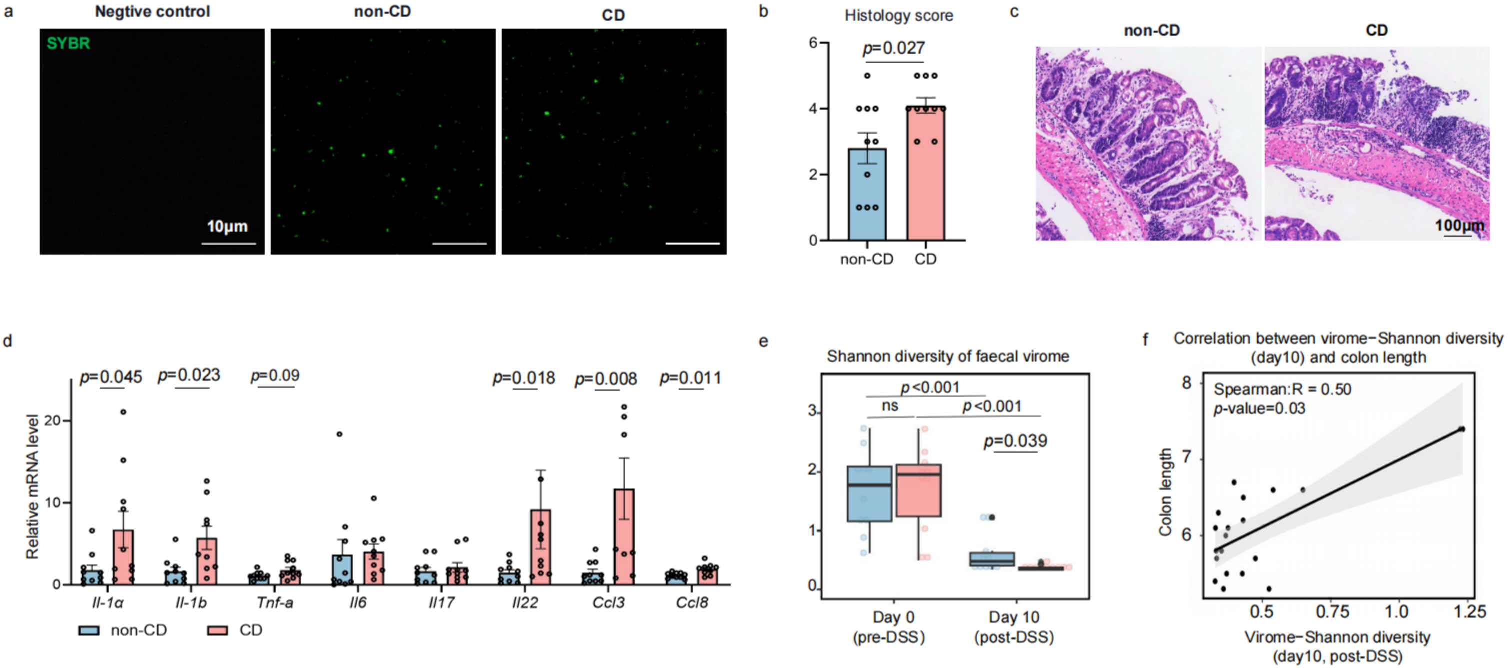

Supplementary Fig. 14 Patient-derived CD ileal VLPs exacerbate intestinal inflammation in mice.
(a) VLP enumeration from human virome preparations. Confocal images of VLPs derived from surgically resected ileal specimens of non-CD or CD patients, stained by SYBR GOLD. SM buffer was used as the negative control.
(b-c) Histology score (b) and representative haematoxylin-eosin-stained intestinal sections (c) of non-CD-VLPs-administered and CD-VLPs-administered mice (n=10 in each group) after DSS treatment (at day 10). Statistical significance was determined by a two-tailed *Mann-Whitney* test, representing as mean +/- S.E..
(d) mRNA expression levels of *Il-1α*, *Il-1β*, *Tnf-α*, *Il 6*, *Il 17*, *Il 22*, *Ccl 3* and *Ccl8* in colonic tissues from non-CD-VLPs-administered and CD-VLPs-administered mice (n=10 in each group), measured by q-PCR. Statistical significance was determined by a two-tailed *Mann-Whitney* test, representing as mean +/- SEM.
(e) The Shannon diversity of faecal virome in non-CD-VLPs-administered and CD-VLPs-administered mice at pre-DSS (on day 0) and post-DSS treatment timepoints (on day 10). For box plots, the boxes extend from the 1st to the 3rd quartile (25th to 75th percentiles), with the median depicted by a horizontal line. Between-group comparison was conducted by a two-tailed Two-way ANOVA test with Sidak's multiple comparisons test, with 10 independent animals in each group.
(f) Correlation between colon length and Shannon diversity of faecal virome at post-DSS timepoint (on day10), calculated via Spearman correlation analysis.

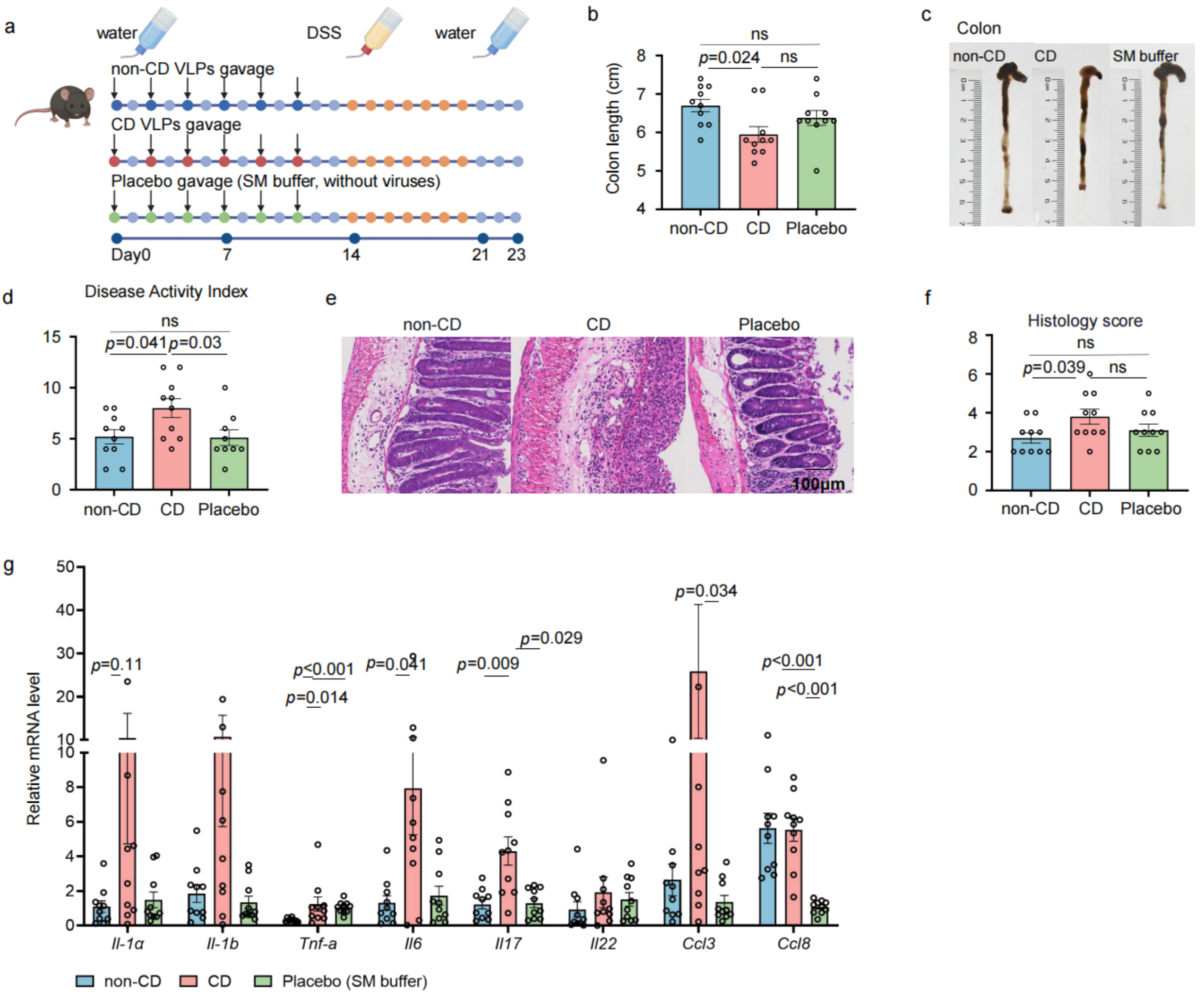

Supplementary Fig. 15 CD ileal VLPs exacerbated intestinal inflammation in DSS-treated mice, a replication study on another batch of mice.

(a)Schematic of animal groupings and treatments (n=10 in each group).

(b-f) Comparisons of colon lengths (b-c), Disease Activity Indexes (d), histology scores and images (e-f) in different groups of mice. Between-group comparison was conducted by one-way ANOVA test, and the plot represented as mean +/- S.E..

(g) Colonic mRNA expression levels of *Il-1α*, *Il-1β*, *Tnf-α*, *Il 6*, *Il 17*, *Il 22*, *Ccl 3* and *Ccl8* in different groups of mice, quantified by q-PCR. Between-group comparison was conducted by one-way ANOVA test, representing as mean +/- S.E..

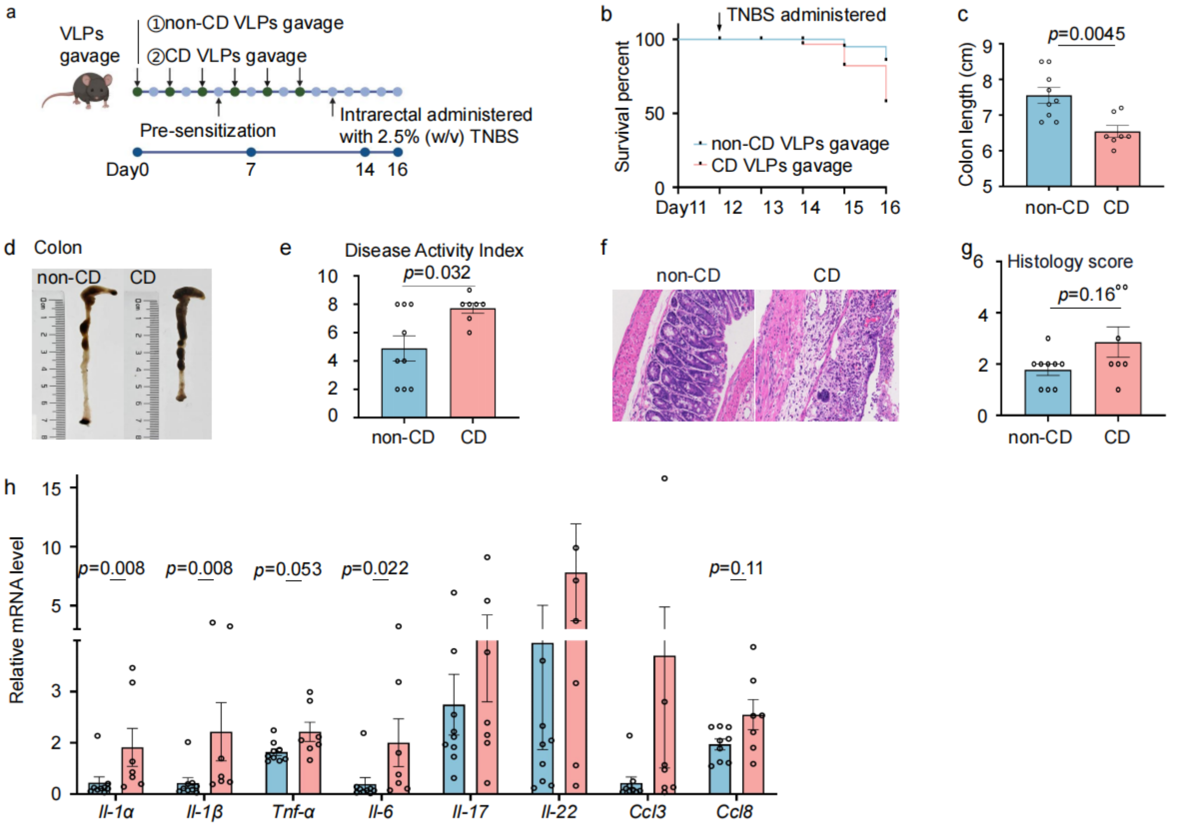

Supplementary Fig. 16 CD ileal VLPs exacerbated intestinal inflammation in TNBS mouse model.

(a) Schematic of animal groupings and treatments (n=10 in each group).

(b) Survival rates of mice from non-CD-VLPs-administered and CD-VLPs-administered group after intrarectal TNBS administration, between-group comparison was conducted by Log-rank test, *p*=0.07.

(c-g) Comparisons of colon lengths (c& d), Disease Activity Indexes (d), histology scores and images (f& g) in different groups of mice. Between-group comparison was conducted by two-tailed *Mann-Whitney* test.

(h) Colonic mRNA expression levels of *Il-1α*, *Il-1β*, *Tnf-α*, *Il 6*, *Il 17*, *Il 22*, *Ccl3* and *Ccl8* in different groups of mice, quantified by q-PCR. Between-group comparison was conducted by two-tailed *Mann-Whitney* test.

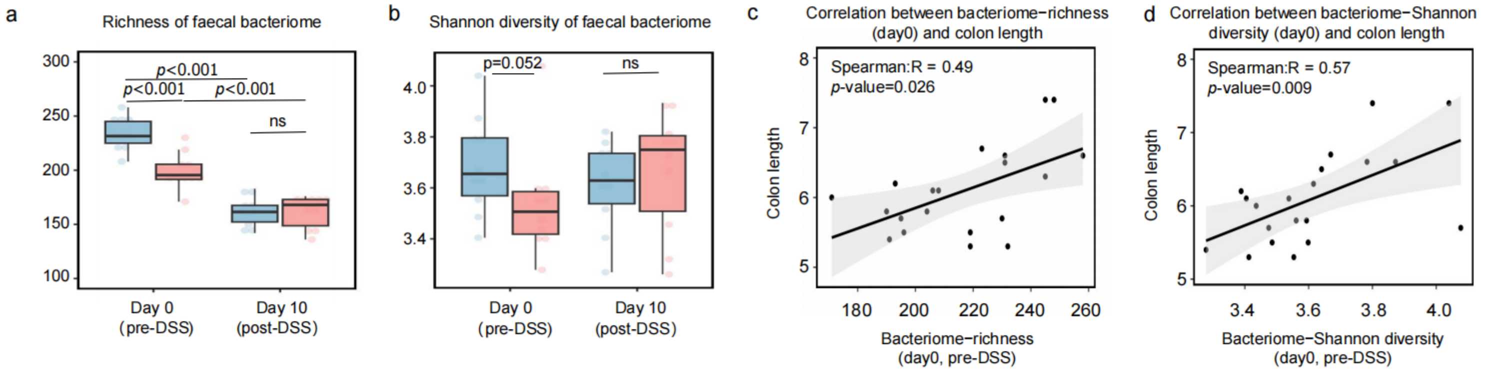

Supplementary Fig. 17 The gut bacteriome reshaped by CD ileal VLPs and its association with severity of intestinal inflammation.

(a-b) The richness (a) and Shannon diversity (b) of faecal bacteriome in non-CD-VLPs-administered and CD-VLPs-administered mice at pre-DSS (at day 0) and post-DSS timepoints (at day 10). For box plots, the boxes extend from the 1st to the 3rd quartile (25th to 75th percentiles), with the median depicted by a horizontal line. Between-group comparison was conducted by a two-tailed Two-way ANOVA test with Sidak's multiple comparisons test, with n=10 mice in each group.

(c-d) Correlation between colon length and richness (c) or Shannon diversity (d) of faecal bacteriome at pre-DSS timepoint (at day 0), calculated via Spearman correlation analysis.

Supplementary Table 1. Quantitative RT-PCR primer

Target gene	Forward primer	Reverse primer
Mice		
mouse-*Gapdh*	CATCACTGCCACCCAGAAGACTG	ATGCCAGTGAGCTTCCCGTTCAG
mouse-*β-actin*	CGTTGACATCCGTAAAGACC	AACAGTCCGCCTAGAAGCAC
mouse-*Il6*	TGGGAAATCGTGGAAATGAG	CCAGTTTGGTAGCATCCATCA
mouse-*Il-1α*	CGAAGACTACAGTTCTGCCATT	GACGTTTCAGAGGTTCTCAGAG
mouse-*Il-1β*	GGATGAGGACATGAGCACCT	AGCTCATATGGGTCCGACA
mouse-*Il17*	TTTAACTCCCTTGGCGCAAAA	CTTTCCCTCCGCATTGACAC
mouse-*Il22*	ATGAGTTTTTCCCTTATGGGGAC	GCTGGAAGTTGGACACCTCAA
mouse-*Tnf α*	GGTGCCTATGTCTCAGCCTCTT	GCCATAGAACTGATGAGAGGGA G
mouse-*Ccl3*	GGCATTCAGTTCCAGGTCAG	TCCCAGCCAGGTGTCATTT
mouse-*Ccl8*	GGGTGCTGAAAAGCTACGAGAG	GGATCTCCATGTACTCACTGACC
Bacteriophage		
Brevibacillus phage Sundance	GCACGTGCTTCCTTCCAATG	TTTGTTCCCCAAGTGCTGGT
Clostridium phage PhiS63	CAGTCCCAGACATTGAGCTT	AGCTGTCATGTATGGATTAGGTC T
Coetzeevirus JL1	CCGACTGCTTTTGGTAACCC	AATCCCAATCGTGCTCGTGT
Lactobacillus phage Lj928	TGGCTCTTTTAGGGATGGTGAC	TTGTAAGTTTCAATTCCAGCATCG
Lughvirus lugh	TCGACCCACCCATCAGC	TCGGCCAGCACTACGGA
Skunavirus E1127	ACGTACATGGCACGAGAATGA	CTTCCGTTGCAACATGCACT
Sozzivirus S13	CGCCGTTGTGCTAATGATGA	CTGCCGTCCATTCAGGTGT
Behunavirus BH1	GCATATTCGTCAACCACCGC	TAAGGATCGACTTCGCGCTC
Aguilavirus mEp043	GCCTTGTCGGTTTCTTCGTG	ACCAAAGTCACTGGCTGCTT
