# Supplementary material for: The gut ileal mucosal virome is disturbed in patients with Crohn’s disease and exacerbates intestinal inflammation in mice
Source: Nat Commun. 2024 Feb 22;15:1638. doi: 10.1038/s41467-024-45794-y (PMC10884039; doi:10.1038/s41467-024-45794-y)
Supplement: Supplementary file 3 — Description of Additional Supplementary Files [file 41467_2024_45794_MOESM3_ESM.docx]

**Supplementary data 1 Clinical metadata**

Summarizing the detailed clinical metadata for each individual participant who enrolled in our study from both Guangzhou and Kunming

**Supplementary data 2 Bacteriophages and bacterial host depleted in both Guangzhou and Kunming CD group versus HC group**

Conclusion of notable depletion of bacteriophages and their corresponding bacterial hosts in the CD group from both Guangzhou and Kunming, when compared with healthy control group.
