# Supplementary material for: The gut ileal mucosal virome is disturbed in patients with Crohn’s disease and exacerbates intestinal inflammation in mice
Source: Nat Commun. 2024 Feb 22;15:1638. doi: 10.1038/s41467-024-45794-y (PMC10884039; doi:10.1038/s41467-024-45794-y)
Supplement: Supplementary file 5 — Supplementary data 2 [file 41467_2024_45794_MOESM5_ESM.pdf]

Supplementary data 2.Bacteriophages and bacterial host depleted in both Guangzhou and Kunming CD group versus HC group

Depleted bacteriophages shared by both Guangzhou and Kunming	Bacterial host	Taxonomic classification of bacterial host				
		pylum	class	order	family	genus
Brevibacillus phage Sundance	Brevibacillus laterosporus/Brevibacillus	Firmicutes	Bacilli	Bacillales	Paenibacillaceae	Brevibacillus
Lactococcus phage 63301	Lactococcus lactis	Firmicutes	Bacilli	Lactobacillales	Streptococcaceae	Lactococcus
Anamdongvirus LBR48	Levilactobacillus brevis	Firmicutes	Bacilli	Lactobacillales	Lactobacillaceae	Levilactobacillus
Junavirus PL1	Lacticaseibacillus paracasei	Firmicutes	Bacilli	Lactobacillales	Lactobacillaceae	Lacticaseibacillu s
Lacnuvirus LcNu	Lacticaseibacillus rhamnosus	Firmicutes	Bacilli	Lactobacillales	Lactobacillaceae	Lacticaseibacillu s
Streptococcus phage phi3396	Streptococcus dysgalactiae	Firmicutes	Bacilli	Lactobacillales	Streptococcaceae	Streptococcus
Coetzeevirus JL1	Lactobacillus	Firmicutes	Bacilli	Lactobacillales	Lactobacillaceae	Lactobacillus
Sozzivirus S13	Oenococcus oeni	Firmicutes	Bacilli	Lactobacillales	Lactobacillaceae	Oenococcus
Pleetrevirus PLE3	Lacticaseibacillus casei BL23	Firmicutes	Bacilli	Lactobacillales	Lactobacillaceae	Lacticaseibacillu s
Tybeckvirus tv521B	Levilactobacillus brevis	Firmicutes	Bacilli	Lactobacillales	Lactobacillaceae	Levilactobacillus
Lafunavirus LF1	Lactobacillus sp.	Firmicutes	Bacilli	Lactobacillales	Lactobacillaceae	Lactobacillus
Colunavirus CL2	Lacticaseibacillus paracasei	Firmicutes	Bacilli	Lactobacillales	Lactobacillaceae	Lacticaseibacillu s
unclassified Efquatrovirus species	Enterococcus	Firmicutes	Bacilli	Lactobacillales	Enterococcaceae	Enterococcus
Colunavirus iLp1308	Lacticaseibacillus paracasei	Firmicutes	Bacilli	Lactobacillales	Lactobacillaceae	Lacticaseibacillu s
Streptococcus phage EJ-1	Streptococcus pneumoniae	Firmicutes	Bacilli	Lactobacillales	Streptococcaceae	Streptococcus
Listeria phage B025	Listeria monocytogenes	Firmicutes	Bacilli	Bacillales	Listeriaceae	Listeria
Microviridae sp.	Unknown	Unknown	Unknown	Unknown	Unknown	Unknown
Microviridae Fen7918_21	Unknown	Unknown	Unknown	Unknown	Unknown	Unknown
Microviridae Bog9017_22	Unknown	Unknown	Unknown	Unknown	Unknown	Unknown
unclassified Caeruleovirus species	Unknown	Unknown	Unknown	Unknown	Unknown	Unknown
unclassified Pleetrevirus species	Lactobacillus	Firmicutes	Bacilli	Lactobacillales	Lactobacillaceae	Lactobacillus
unclassified Caudoviricetes species	Unknown	Unknown	Unknown	Unknown	Unknown	Unknown

Depleted bacteriophages shared by both Guangzhou and Kunming	Bacterial host	Taxonomic classification of bacterial host				
		pylum	class	order	family	genus
Pleeduovirus PLE2	Lacticaseibacillus casei BL23	Firmicutes	Bacilli	Lactobacillales	Lactobacillaceae	Lacticaseibacillus
Planktothrix phage PaV-LD	Planktothrix agardhii	Cyanobacteria	Oscillator	Oscillatoriales	Microcoleaceae	Planktothrix
Lughvirus lugh	Faecalibacterium	Firmicutes	Clostridia	Eubacteriales	Oscillospiraceae	Faecalibacterium
Likavirus caliburn	Streptomyces griseus	Actinobacteria	Actinomycetia	Streptomycetales	Streptomycetaceae	Streptomyces
Skunavirus E1127	Lactococcus lactis	Firmicutes	Bacilli	Lactobacillales	Streptococcaceae	Lactococcus
unclassified Lubbockvirus species	Clostridium	Firmicutes	Clostridia	Eubacteriales	Clostridiaceae	Clostridium
Microviridae Fen418_41	Unknown	Unknown	Unknown	Unknown	Unknown	Unknown
Samwavirus samW	Corynebacterium xerosis	Actinobacteria	Actinomycetia	Corynebacteriales	Corynebacteriaceae	Corynebacterium
Eponavirus epona	Faecalibacterium	Firmicutes	Clostridia	Eubacteriales	Oscillospiraceae	Faecalibacterium
Lactobacillus phage Lv-1	Lactobacillus jensenii	Firmicutes	Bacilli	Lactobacillales	Lactobacillaceae	Lactobacillus
Punavirus SJ46	Salmonella enterica serovar Indiana	Proteobacteria	Gammaproteobacteria	Enterobacterales	Enterobacteriaceae	Salmonella
Junavirus LJ	Lacticaseibacillus casei W56	Firmicutes	Bacilli	Lactobacillales	Lactobacillaceae	Lacticaseibacillus
