# Supplementary material for: The gut ileal mucosal virome is disturbed in patients with Crohn’s disease and exacerbates intestinal inflammation in mice
Source: Nat Commun. 2024 Feb 22;15:1638. doi: 10.1038/s41467-024-45794-y (PMC10884039; doi:10.1038/s41467-024-45794-y)
Supplement: Supplementary file 6 — Reporting Summary [file 41467_2024_45794_MOESM6_ESM.pdf]

nature portfolio

Corresponding author(s): Tao Zuo

Last updated by author(s): Jan 25, 2024

Reporting Summary

Nature Portfolio wishes to improve the reproducibility of the work that we publish. This form provides structure for consistency and transparency in reporting. For further information on Nature Portfolio policies, see our Editorial Policies and the Editorial Policy Checklist.

Statistics

For all statistical analyses, confirm that the following items are present in the figure legend, table legend, main text, or Methods section.

n/a	Confirmed	
☐	☒	The exact sample size (*n*) for each experimental group/condition, given as a discrete number and unit of measurement
☐	☒	A statement on whether measurements were taken from distinct samples or whether the same sample was measured repeatedly
☐	☒	The statistical test(s) used AND whether they are one- or two-sided<br>*Only common tests should be described solely by name; describe more complex techniques in the Methods section.*
☐	☒	A description of all covariates tested
☐	☒	A description of any assumptions or corrections, such as tests of normality and adjustment for multiple comparisons
☐	☒	A full description of the statistical parameters including central tendency (e.g. means) or other basic estimates (e.g. regression coefficient) AND variation (e.g. standard deviation) or associated estimates of uncertainty (e.g. confidence intervals)
☐	☒	For null hypothesis testing, the test statistic (e.g. *F*, *t*, *r*) with confidence intervals, effect sizes, degrees of freedom and *P* value noted<br>*Give P values as exact values whenever suitable.*
☒	☐	For Bayesian analysis, information on the choice of priors and Markov chain Monte Carlo settings
☒	☐	For hierarchical and complex designs, identification of the appropriate level for tests and full reporting of outcomes
☐	☒	Estimates of effect sizes (e.g. Cohen's *d*, Pearson's *r*), indicating how they were calculated

Our web collection on statistics for biologists contains articles on many of the points above.

Software and code

Policy information about availability of computer code

Data collection	No software was used for data collection.
Data analysis	Virome profiling was generated via the following pipelines or algorithms: Trimmomatic (v0.39); KneadData (v0.7.4); MEGAHIT (v1.2.9); VirSorter2 (v2.2.3); DeepVirFinder; CAT (v5.2.3); BUSCO (v5.3.2); Prodigal (v2.6.3); vContact2 (v0.11.3); diamond (v2.0.11); Bowtie2 (v2.3.5.1); BBMap (v38.84); blastp (v2.12.0). The functionality of ileal virome were analyzed via prokka (v1.12); diamond (v2.0.11) and Bowtie2 (v2.3.5.1). Bacteriome profiling were analyzed by Qiime2 (v2022.2.0). The lifestyle of bacteriophages were analyzed via PhaTYP, while the bacterial host prediction of phages were performed by PHYBOX (CHERRY). The correlations between abundance/diversity of bacteriome and virome were analyzed via FastSpar (v1.0.0) and ggcor respectively. Feature taxa associated with metadata variables (HC versus CD, intestinal inflammation, medications, and dietary factors) were identified using MaAsLin2; Covariates of virome/bacteriome were identified using vegan package in R. Metagenomic data analysis for bacteriome profiling was analyzed using metaphlan4 (v4.0.4).Transcriptomics sequencing (RNA-seq) data was analyzed with HISAT2HISAT2, whilst the differentially expressed gene (DEG) analysis were performed with DESeq2. Multi-omics analysis and microbiome factors identification were conducted by MOFA. All figures were visualized by RStudio, R (v4.1.3) and GraphPad Prism 8.<br>Please ensure all the data collection/data analysis software/tools/algorithms/packages used in the study are clearly mentioned in the manuscript and are also listed here in the reporting summary (with version numbers).<br>The code utilized in this study has been made publicly available at the following GitHub repository: https://github.com/ouczt/Crohn_disease_virome_Zuotao_Lab, and it can also be accessed via DOI: 10.5281/zenodo.10538381.

For manuscripts utilizing custom algorithms or software that are central to the research but not yet described in published literature, software must be made available to editors and reviewers. We strongly encourage code deposition in a community repository (e.g. GitHub). See the Nature Portfolio guidelines for submitting code & software for further information.

Data

Policy information about availability of data

All manuscripts must include a data availability statement. This statement should provide the following information, where applicable:

- Accession codes, unique identifiers, or web links for publicly available datasets
- A description of any restrictions on data availability
- For clinical datasets or third party data, please ensure that the statement adheres to our policy

The raw metagenomic sequencing data for virome profiling and 16S rDNA sequencing data for bacteriome profiling have been deposited in the Genome Sequence Archive (GSA) in National Genomics Data Center (http://ngdc.cncb.ac.cn), Beijing institute of Genomics (BIG), Chinese Academy of Sciences, under accession HRA005245 (Bioproject: PRJCA018565, for controlled access to abide by the Human Genetic Resources Administration of China regulation. The raw transcriptomics sequencing (RNA-seq) data have been deposited in the Genome Sequence Archive in National Genomics Data Center under accession CRA011968 (Bioproject: PRJCA018565). The raw metagenomic sequencing data for animal experiment have been deposited in the Genome Sequence Archive (GSA) in National Genomics Data Center, under accession CRA012045 (Bioproject: PRJCA018565). In accordance with the China government's policy and regulations on human genetic materials for the Chinese population, all data reported in this paper shall be shared by the lead contact upon request and joint regulatory approval from the Ministry of Science and Technology of China, Sun Yat-sen University, and Kunming Medical University.

In addition, the following public databases were also used in this paper: Virus-Host DB (https://www.genome.jp/virushostdb/note.html), Uniprot knowledgebase database (release 2022_04, https://www.uniprot.org/help/uniprotkb), SILVA SSU database (SILVA 138 SSU Ref NR 99, https://www.arb-silva.de/), RefSeq-v212 database (https://www.ncbi.nlm.nih.gov/refseq/), and BUSCO database (bacteria_odb10, https://busco.ezlab.org/) and human genome reference database (GRCh38 p12, https://www.ncbi.nlm.nih.gov/datasets/genome/).

Research involving human participants, their data, or biological material

Policy information about studies with human participants or human data. See also policy information about sex, gender (identity/presentation), and sexual orientation and race, ethnicity and racism.

Reporting on sex and gender	The term 'sex' was used throughout the study. The study included a total of 208 individuals based on a random selection, of which 63.4% individuals are male. Sex was determined based on self-reporting. Sex and gender were not considered in recruitment of patients, nor in selection of sample to be sequenced, while age- and sex-matched healthy controls were recruited subsequently. Our primary findings did not apply to only one sex, and the further analysis considered sex as a potential covariates and results showed that it showed no significant effect on ileal virome composition.
Reporting on race, ethnicity, or other socially relevant groupings	Not applicable.
Population characteristics	A totally of 208 individuals from Guangzhou or Kunming were enrolled in our study, consisting of 105 healthy individuals and 103 CD patients , with a average age of 35.90 (interquartile range, 28-45 years) and 33.90 (interquartile range, 25-41.5 years), respectively.
Recruitment	In our study, we recruited a total of 208 participants, including CD patients and HC, from the Six Affiliated Hospital of Sun Yat-sen University in Guangzhou, China, and The First Affiliated Hospital of Kunming Medical School in Kunming, China.<br>We used a multi-stage process to recruit participants for our study. Initially, potential participants were identified based on their CD diagnosis. These individuals were then screened according to our inclusion and exclusion criteria, which were designed to ensure that our participant pool was representative of the population we aimed to study. Patients who met our eligibility criteria were first included as potential candidates. If they agreed to participate, the study was explained in detail and informed consent was obtained. It's important to note that our participants were not randomly selected, so our sample may not fully represent the broader population, potentially leading to selection bias.<br>Additionally, age- and sex-matched healthy individuals were commercially recruited from these two centers. This could result in a self-selected population that differs from the CD group in terms of demographics, environmental factors, and lifestyles, potentially leading to self-selection bias. Despite our efforts to control for these variables, some may still impact our results. There may also be variables that we did not account for in our study that could influence the outcomes, potentially leading to confounding bias.
Ethics oversight	The study was approved respectively by the Institutional Review Board (IRB) of the Research Ethics Committee of the Six Affiliated Hospital of Sun Yat-sen University (Ref. No: 2021ZSLYEC-245) and the IRB of Research Ethics Committee of the First Affiliated Hospital of Kunming Medical School (Ref. No: 2022.L.94). In accordance with ethical guidelines, informed consent was obtained for all participants in this study. For participants under the age of 18, informed consent was obtained from their legally authorized representatives (LARs).

Note that full information on the approval of the study protocol must also be provided in the manuscript.

Field-specific reporting

Please select the one below that is the best fit for your research. If you are not sure, read the appropriate sections before making your selection.

☒ Life sciences ☐ Behavioural & social sciences ☐ Ecological, evolutionary & environmental sciences

For a reference copy of the document with all sections, see nature.com/documents/nr-reporting-summary-flat.pdf

Life sciences study design

All studies must disclose on these points even when the disclosure is negative.

Sample size	No statistic method was used to predetermined the sample size. Sample saturation curve analyses based on the ileal mucosal virome at both the family and species levels showed that the current sample size (n=208) of our study was sufficient to capture the overall virome diversity (Supplementary figure 1B) and hence allowed us to compare the configurational difference between HC and CD. Sample sizes for animal studies were determined based on previous results for similar in vivo experiments (PMID: 32165408, PMID: 35799219, PMID: 30763538).
Data exclusions	No experimental data was excluded.
Replication	Illumina sequencing was performed once per sample due to the extremely limited sample volume and low concentration of DNA in the samples. All DNA has been utilized in the preparation of sequencing libraries. In addition, we conducted two rounds of animal experiments in the DSS model and verified the pro-inflammatory phenotype of CD mucosal VLPs in the TNBS model. We conducted our in vitro experiments with at least three replicates, and the attempts at replication were successful via using fecal samples from five distinct individuals. Our in vivo experiments were specifically designed to confirm the conclusions drawn from our bioinformatics analysis. To comply with the ethical guidelines from IACUC at the Sun Yat-sen University advocating for the reduction of animal uses and welfare of animals, we replicated the essential animal experiments to validate our primary findings that substantiated the causal effect of virome in inflammation but not for those experiments involving a further large number of animals.
Randomization	Our study in human was a cross-sectional, case-control observational study without any treatments or interventions, we therefore didn't apply randomization and instead included consecutive patients who met the eligibility criteria. For controlling covariates, age- and sex-matched individuals were recruited from two centers. For controlling for other potential confounders, we adopted multivariate adjustment in our analyses where appropriate. In animal experiment, all animals were randomly allocated into groups, with 10 mice in each group.
Blinding	Investigators were blinded to group allocation and all demographic information during sputum sample processing and sequencing, while the collection and final data analysis was performed unblinded as all samples were labeled to ensure accuracy and the final analysis required grouping patients by disease severity. In animal experiment, all experimental techniques and severity of intestinal inflammation evaluation were performed blinded with the samples coded and randomized. The key for the codes was only broken after data acquisition to perform final analysis.

Reporting for specific materials, systems and methods

We require information from authors about some types of materials, experimental systems and methods used in many studies. Here, indicate whether each material, system or method listed is relevant to your study. If you are not sure if a list item applies to your research, read the appropriate section before selecting a response.

Materials & experimental systems

n/a	Involved in the study
☒	☐ Antibodies
☒	☐ Eukaryotic cell lines
☒	☐ Palaeontology and archaeology
☐	☒ Animals and other organisms
☐	☒ Clinical data
☒	☐ Dual use research of concern
☒	☐ Plants

Methods

n/a	Involved in the study
☒	☐ ChIP-seq
☒	☐ Flow cytometry
☒	☐ MRI-based neuroimaging

Animals and other research organisms

Policy information about studies involving animals; ARRIVE guidelines recommended for reporting animal research, and Sex and Gender in Research

Laboratory animals	6-week-old male C57BL/6J mice were purchased from Beijing Vital River Laboratory Animal Technology Corporation (Beijing, China). All mice were housed in specific pathogen-free conditions. Temperatures of 18-25°C with 50-60% humidity and 14-hour light/10-hour dark cycle are used. Dailed information for animal study can be found in the section of Method.
Wild animals	No wild animals were used in this study.
Reporting on sex	Male mice were used in our experiment.
Field-collected samples	This experiment were not included field-collected samples.
Ethics oversight	The animal experiment was conducted in accordance with the animal use protocol approved by Institutional animal care and use committee (IACUC) of Sun Yat-sen University and in compliance with animal ethical regulations.

Note that full information on the approval of the study protocol must also be provided in the manuscript.

Clinical data

Policy information about clinical studies

All manuscripts should comply with the ICMJE guidelines for publication of clinical research and a completed CONSORT checklist must be included with all submissions.

Clinical trial registration	Not applicable.
Study protocol	Samples were collected following a standard operating procedure, summarised in supplementary materials.
Data collection	Clinical phenotype and metadata of study subjects were obtained by medical practitioners. Dietary questionnaire investigation was conducted by a dietitian. Procedures of endoscopic assessment, ileal biopsy obtainment, and sample storage followed a standardised operation procedure shared between centres.
Outcomes	Not applicable.
